# Iron induces insulin resistance in cardiomyocytes via regulation of oxidative stress

**DOI:** 10.1038/s41598-019-41111-6

**Published:** 2019-03-15

**Authors:** Hye Kyoung Sung, Erfei Song, James Won Suk Jahng, Kostas Pantopoulos, Gary Sweeney

**Affiliations:** 1 Department of Biology, York University, Toronto, Ontario Canada; 2 Lady Davis Institute for Medical Research and McGill University, Montreal, Quebec Canada

## Abstract

Iron overload is associated with various pathological changes which contribute to heart failure. Here, we examined mechanisms via which iron alters cardiomyocyte insulin sensitivity. Treatment of primary adult and neonatal cardiomyocytes as well as H9c2 cells with iron decreased insulin sensitivity determined via Western blotting or immunofluorescent detection of Akt and p70S6K phosphorylation and glucose uptake. Using CellROX deep red or DCF-DA probes we also observed that iron increased generation of reactive oxygen species (ROS), and that pretreatment with the superoxide dismutase mimetic MnTBAP reduced ROS production and attenuated iron-induced insulin resistance. SKQ1 and allopurinol but not apocynin reduced iron-induced ROS suggesting mitochondria and xanthine oxidase contribute to cellular ROS in response to iron. Western blotting for LC3-I, LC3-II and P62 levels as well as immunofluorescent co-detection of autophagosomes with Cyto-ID and lysosomal cathepsin activity indicated that iron attenuated autophagic flux without altering total expression of Atg7 or beclin-1 and phosphorylation of mTORC1 and ULK1. This conclusion was reinforced via protein accumulation detected using Click-iT HPG labelling after iron treatment. The adiponectin receptor agonist AdipoRon increased autophagic flux and improved insulin sensitivity both alone and in the presence of iron. We created an autophagy-deficient cell model by overexpressing a dominant-negative Atg5 mutant in H9c2 cells and this confirmed that reduced autophagy flux correlated with less insulin sensitivity. In conclusion, our study showed that iron promoted a cascade of ROS production, reduced autophagy and insulin resistance in cardiomyocytes.

## Introduction

Iron is an essential micronutrient and its crucial role in many physiological functions is often underestimated^1^. Altered iron metabolism is implicated in a vast array of diseases, including type 2 diabetes^1^, neurodegenerative diseases^2^, cardiovascular diseases^3^, cancer^4^, osteoporosis^5^ and many more. In particular, both iron deficiency (ID) and iron overload (IO) have been associated with cardiomyopathy^3^. Recently, iron overload cardiomyopathy (IOC) has been described as a secondary form of cardiomyopathy resulting from the accumulation of iron in the myocardium mainly because of genetically determined disorders of iron metabolism or multiple transfusions^6^. Iron is a vital structural component of hemoglobin, myoglobin, oxidative enzymes and respiratory chain proteins that are collectively responsible for oxygen transport, storage, and energy metabolism^7^. Iron-overload cardiomyopathy is the most common reason for mortality in patients with secondary iron overload or patients with early onset forms of genetic hemochromatosis^8^.

In essence, altered iron homeostasis leads to uncontrolled iron deposition in different organs, including the heart, leading to progressive tissue damage^8^. Iron-induced oxidative stress plays an important role in the pathogenesis of iron-overload mediated heart disease^9,10^. The formation of labile NTBI alters the pro-oxidant/antioxidant balance, leading to a pro-oxidant state with increased free radical production, oxidative stress and cellular damage^11^. Previous studies indicated that oxidative stress can lead to mitochondrial dysfunction and accumulation of lipotoxic metabolites which have been shown to contribute to insulin resistance^12,13^.

Autophagy is a cellular degradation process capable of clearing damaged mitochondria and protein aggregates^14,15^. Autophagy has been called a double-edged sword as it can have either beneficial or detrimental effects on the heart^16^. Recent evidence indicated that dysregulation of autophagy resulted in ER stress, insulin resistance and glucose intolerance^17^. Our own research also has shown that induction of autophagy can be beneficial to the myocardium in terms of its insulin-sensitizing effect and reduce apoptosis^18^. In various tissue types, it has been found that ROS production results in increased autophagy^19^. In the heart, elevated autophagy is activated post-ischemia in association with ROS upregulation and this is thought to be an endogenous self-protective mechanism^20^. ROS also play an early role in the development of insulin resistance^21^. Evidence suggested that downstream of the PI3K/Akt insulin signaling pathway may be the target of exogenous inducers of autophagy^22^.

The precise molecular mechanisms of iron-overload cardiomyopathy have not been elucidated yet. In this study, we hypothesized that iron induces insulin resistance in cardiomyocytes and that this involves regulation of autophagy and/or oxidative stress and crosstalk between them. To do so, we used primary adult or neonatal cardiomyocytes and H9c2 cells as cellular models and treated with iron for up to 24 h and tested ROS production, autophagic flux, and insulin sensitivity.

## Results

### Systemic administration of iron induced insulin resistance in mice

**Figure 1:**
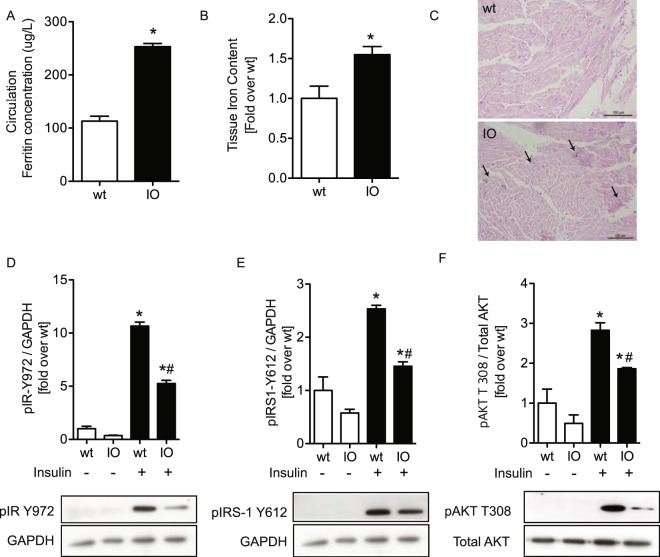
Iron overload significantly induced insulin resistance. Characterizing intracellular iron by serum ferritin (**A**), Colorimetric assay (**B**) and Perl’s Prussian blue staining (**C**) from wt and IO mice. Western blots showing phosphorylation of IR Tyr 972, (**D**) IRS-1 Tyr 612 (**E**) and AKT T308 (**F**) and the reference protein, GAPDH and total AKT, in heart tissue lysates from wt and IO mice. The images were collected from two gels with same loading amounts. The blot gels shown are representatives of three different experiments (n = 3). *Indicates significant difference from wt without insulin *p < 0.05, ^#^indicates significant difference from wt with insulin ^#^p < 0.05, Scale bar PB = 100 μm.

We first generated an animal model in which injection of iron caused a reduction in myocardial insulin sensitivity after 24 hr. Mice were injected with iron dextran at 15 mg per kg via tail vein three times, with two hours intervals, to induce iron overload. As expected, the ferritin content of plasma was significantly greater in the iron overload (IO) group, than wild type (wt) group (Fig. 1A). Using a ferrozine-based assay to detect intracellular iron in heart homogenates (Fig. 1B) and Perl’s Prussian blue staining of cardiac tissue sections (Fig. 1C), we found that there was a small but significant increase in iron accumulation in IO mice. We then injected insulin via tail vein to determine cardiac sensitivity to insulin. Our data indicated reduced insulin-stimulated phosphorylation of insulin receptor (Y972), insulin receptor substrate-1 (Y612) and Akt (T308) after iron overload (Fig. 1D–F).

### Intracellular iron accumulation induced insulin resistance in H9c2 cells

**Figure 2:**
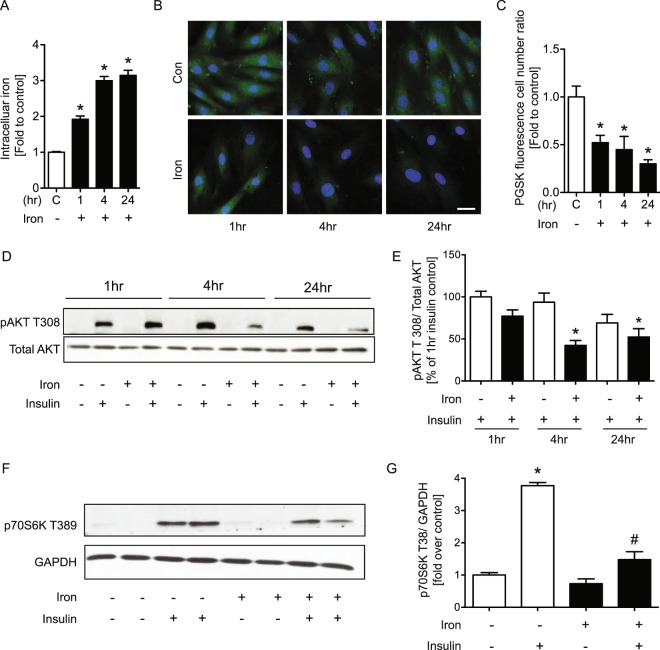
Intracellular iron accumulation induced insulin resistance in cardiomyocyte. H9c2 cells were treated with FeSO_4_ (100 μM) or without (con) for 1, 4 and 24 hrs. Colorimetric assay (**A**) and PGSK assay (**B**) showing intracellular iron, followed by its quantification (**C**). H9c2 cells without (con) or with FeSO_4_ (100 μM) were treated with insulin (100 nM) for 10 minutes before experimental endpoint. Western blots showing phosphorylation of AKT T308 and the reference protein, total AKT (**D**), phosphorylation of p70S6K T389 and the reference protein, GAPDH (**F**), and their quantifications (**E**,**G**). The images were collected from different parts of the same gel with same loading amounts. The blot gels shown are representatives of five different experiments (n = 5) *indicates significant difference from control *p < 0.05, Scale bar = 20 μm.

We next used an *in vitro* model of iron overload in the H9c2 cardiomyoblast cell line derived from rat ventricle. After treatment with iron for 1, 4 and 24 hr a colorimetric intracellular iron assay was conducted by adding ferrozine to H9c2 cell lysates (Fig. 2A). In addition, the fluorescent dye PGSK which is quenched by iron was preloaded into cells 30 minutes before iron treatment and compared to cells cultured for the same time period without iron treatment (Fig. 2B,C). Both assays showed a time-dependent increase in intracellular iron accumulation from 1 to 24 hr. We next examined the effects of iron on insulin sensitivity in these cells via Western blotting detection of Akt phosphorylation and the data indicated that the response to insulin was significantly attenuated after 4 or 24 hr iron overload (Fig. 2D,E). We further confirmed that iron blunted insulin signalling by detection of p70S6K phosphorylation (Fig. 2F,G).

### Iron overload induced insulin resistance in primary adult and neonatal rat cardiomyocytes

**Figure 3:**
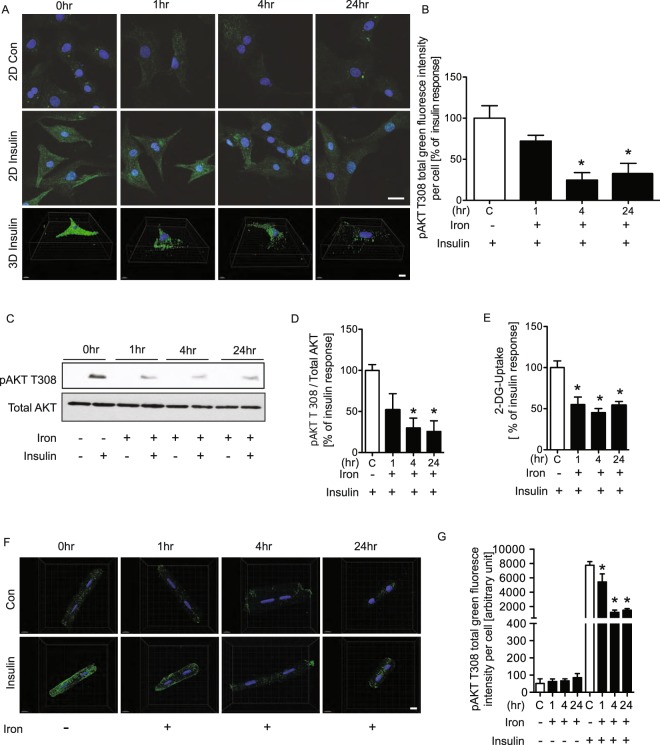
Insulin signaling in cardiomyocyte indicated by increased phosphorylation of AKT T308 was all decreased by iron. Primary adult and neonatal cardiomyocytes with FeSO_4_ (100 μM) or without (con) were treated with insulin (100 nM) for 10 minutes before experimental endpoint. Representative confocal images of phosphorylation of AKT T308 in neonatal (**A**) and adult cardiomyocyte cells (**F**) and their quantifications (**B**,**G**). Western blots showing phosphorylation of AKT T308 and the reference protein, total AKT, in neonatal cardiomyocyte cells (**C**) its quantification (**D**). The images were collected from different parts of the same gel with same loading amounts. The blot gels shown are representatives of five different experiments (n = 5). Representative graph of the examined effect of FeSO_4_ (100 uM) for 1, 4, and 24hrs on glucose uptake (**E**). *Indicates significant difference from control without insulin; ^#^Indicates significant difference from control respective to insulin 100 nM. *p < 0.05, Scale bar = 20 μm, (**A**,**D** n = 3, E n = 5, **F,G** n = 3).

After our initial observation that iron induced insulin resistance in H9c2 cells, we examined whether the same phenomenon was observed in primary cardiomyocytes. First, we isolated primary neonatal rat cardiomyocytes and observed that after 4 and 24 hr iron treatment there was a decrease in Akt phosphorylation detected by immunofluorescence (Fig. 3A,B) and Western blotting (Fig. 3C,D). In addition, the metabolic significance of these effects was examined by measuring glucose uptake in primary neonatal rat cardiomyocytes. We observed that iron significantly attenuated insulin-stimulated glucose uptake (Fig. 3E). Furthermore, we then isolated primary adult rat cardiomyocytes and used immunofluorescent detection of Akt phosphorylation to show that insulin signaling was significantly attenuated by iron treatment in these cells (Fig. 3F,G).

### Iron induced ROS production in H9c2 cells

**Figure 4:**
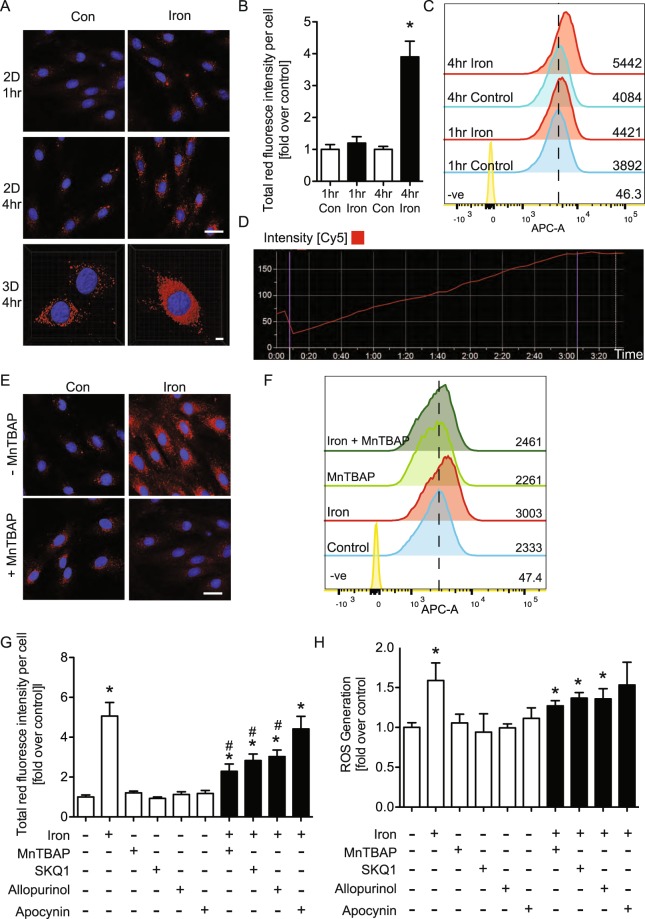
Iron increased generation of reactive oxygen species (ROS) and enhanced by antioxidant (MnTBAP, 100 μM). H9c2 cells with FeSO_4_ (100 μM) or without (con) were treated at 1 and 4hrs by CellROX Red assay and imaged by confocal microscope (**A,E**). Total red fluoresce of the confocal image was quantified and graphed (**B**) and shown in a time-lapse graph in live cells (**D** & Supplementary Video). The reactive oxygen species (ROS) production was examined by CellROX Red assay using flow cytometry (**C**,**F**) the high content system (**G**) and DCF-DA assay (**H**). *Indicates significant difference from control *p < 0.05, ^#^Indicates significant difference from iron ^#^p < 0.05, Scale bar = 20 μm, n = 4.

We next investigated the effect of iron on intracellular ROS production. This was first performed using the ROS indicator dye CellROX^®^ red, and the representative 2- and 3-dimensional fluorescent images in Fig. 4A and quantitation in B showed that appearance of ROS was increased upon iron treatment at 4 hr. A detailed quantitative and temporal study using live cell imaging is shown in Fig. 4D (also see Supplementary Video File) and this clearly indicated that ROS production reached a maximum after 3 hr and plateaued thereafter. The significant increase in intracellular ROS at 4 hr was further confirmed by detecting CellROX^®^ red signal via flow cytometry (Fig. 4C). As expected, we found using fluorescent confocal imaging or flow cytometry that pretreatment of cardiomyocytes with MnTBAP, a superoxide dismutase (SOD) mimetic^23^, attenuated the overall increase in ROS after 4 hr iron exposure (Fig. 4E,F). To further investigate the source of ROS which was stimulated by iron, we pretreated in H9c2 cells with SkQ1, a specific inhibitor of mitochondrial ROS production^24^; allopurinol, a specific xanthine oxidase inhibitor^25,26^ and apocynin, a specific NADPH oxidase inhibitor^27^. Both immunofluroscent images using CellROX^®^ red and quantitative analysis of ROS production detected using DCFDA showed that iron induced ROS production was attenuated by SKQ1 and allopurinol but not apocynin (Fig. 4G,H).

### Iron inhibited autophagy and reduced protein clearance in H9c2 cells

**Figure 5:**
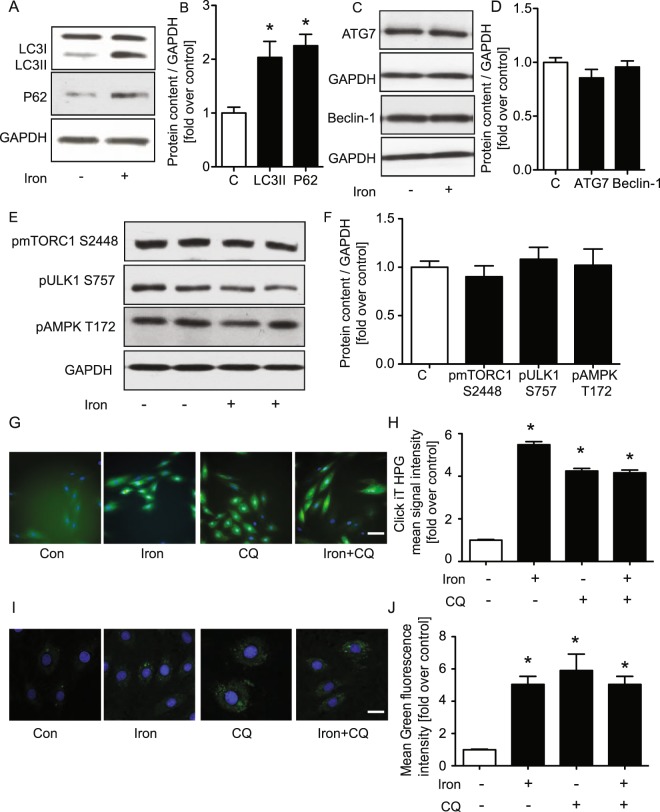
Regulation of protein clearance by inhibition of autophagy and effect of iron. H9c2 cells were treated ± FeSO_4_ (100 μM) and ± chloroquine (60 nM) for 4 hrs. Representative western blotting for autophagy-related genes (LC3II, P62, ATG7, Beclin-1, pmTORC1 S2448, pULK1 S757 and pAMPK T172) and the reference protein, GAPDH is shown (**A,C,E**). Corresponding quantifications of western blots are shown (**B,D,F**). Detection of protein synthesis/clearance was measured using Click-iT HPG Alexa Fluor 488 kit, and imaged by CX7 high content analysis microscopy from which representative images (**G**) and quantification are shown (**H**). Autophagosome detection using Cyto ID fluorescent probe and confocal microscopy is shown (**I**) and quantified (**J**). *Indicates significant difference from control *p < 0.05, ^#^Indicates significant difference from iron ^#^p < 0.05, Scale bar = 50 (G) and 20 (I) μm, n = 4.

We next examined the potential mechanistic role of autophagy in regulating the effects of iron and first confirmed that at 4 hr of iron treatment, an increased amount of LC3-II and P62 was observed (Fig. 5A,B). When autophagic flux pathway is complete, autophagosomes will fuse with lysosome to get degraded. Interestingly, iron had no direct effect on expression of autophagy related proteins Atg7 and beclin-1 (Fig. 5C,D), or on phosphorylation of mTORC1 S2448, ULK1 S757 and AMPK T172 (Fig. 5E,F). We next employed a non-radioactive, metabolic Click-iT HPG Alexa Fluor 488 labelling assay to quantify protein synthesis or degradation^28^ and examined effect of 4 hr iron in the presence or absence of chloroquine. Chloroquine-mediated blockade of the autophagy causes an accumulation of protein which is also observed after 4 hr iron treatment, and their effects were not additive (Fig. 5G,H). To further determine the direct effect of iron on autophagy, we then used Cyto ID (green) to detect autophagosomes and found a significantly increased autophagosome accumulation at 4 hr iron treatment or, as expected, in the presence of chloroquine (Fig. 5I,J).

### The mechanism via which iron reduced insulin signaling is via inhibition of autophagy

**Figure 6:**
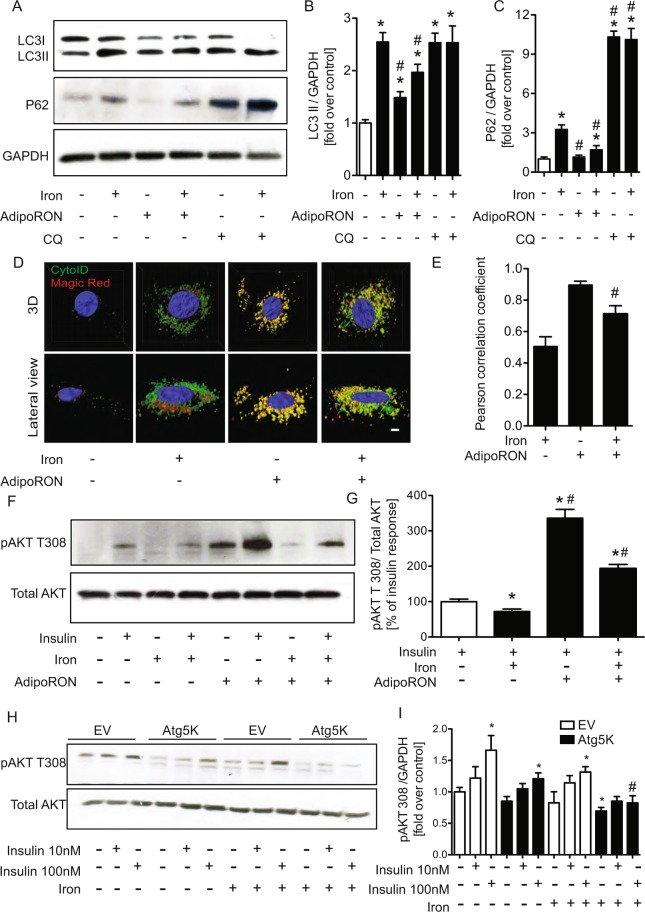
Regulation of insulin signaling by alterations in autophagy and effect of iron. H9c2 cells were treated with FeSO4 (100 μM) ± adipoRON (35 μM) and ± chloroquine (60 nM) for 4 hrs. Western blots showing protein expression of LC3II, P62 and the reference protein GAPDH, and their quantifications (**B**,**C**). Increase in autophagy by iron was validated by co localization of Cyto ID with Capthepsin D magic red assay (**D**) using confocal microscope and quantified (**E**). Insulin sensitivity was decreased in H9c2 cells as indicated by decreased phosphorylation of Akt T308. Western blots show phosphorylation of AKT T308 and the reference protein, total Akt (**F**), followed by their quantifications (**G**). Phosphorylation of AKT (T308) in autophagy-impaired Atg5K cells is shown as representative western blot (**H**) and quantification (**I**). The images shown were collected from different regions of the same gel and are representatives of n = 5. *Indicates significant difference from control *p < 0.05, ^#^Indicates significant difference from iron ^#^p < 0.05, Scale bar = 20 μm, and in A–G n = 4.

We examined the potential mechanistic role of autophagy in regulating effects of iron on insulin sensitivity. Autophagy flux was first analyzed via Western blotting for LC3 and p62 ± chloroquine and data indicated that iron inhibited autophagy flux (Fig. 6A–C). We also used AdiopoRON, an adiponectin receptor agonist, as a means to stimulate autophagy and indeed observed increased LC3-II levels without an increase in p62 (Fig. 6A–C). To further determine the effect of iron on autophagic flux, we then used Cyto ID (green) to detect autophagosomes together with Magic Red (red) co-localization. Magic Red fluorescence indicates activity of the lysosomal cysteine protease cathepsin B^29^. Figure 6D,E shows that AdipoRon promoted co-localization of these signals which appear as yellow puncta, indicating increased autophagic flux, whereas distinct red and green signals were evident after iron treatment. Iron decreased Cyto ID with Magic Red co-localization yellow puncta in cardiomyocytes. Addition of AdipoRon together with iron restored autophagic flux. Taken together, data indicate that iron inhibited overall autolysosomal activity and hindered autophagic flux. Figure 6F,G shows that, as expected, AdipoRon significantly increased the basal and insulin-stimulated phosphorylation of Akt and more interestingly could significantly recover the inhibitory effect of iron on insulin signaling. These data suggest that insulin resistance caused by iron could be restored by rescuing the inhibition of autophagy. Based on these observations we generated a stable cell line with autophagy deficiency induced by overexpression of a dominant-negative Atg5 mutant ATG5K130R^30^. With the use of autophagy deficient cells, there was an attenuation of dose dependent insulin-stimulated phosphorylation of Akt Thr308 compared to cells transfected with empty vector (Fig. 6H,I), indicating the requirement for adequate autophagic flux in maintaining insulin sensitivity. The ability of iron to induce insulin resistance was maintained in autophagy deficient cells, and a slightly enhanced effect was observed in response to 100 nM insulin (Fig. 6H,I).

### Iron induced insulin resistance and reduction in autophagy flux occurred downstream of increased ROS production

**Figure 7:**
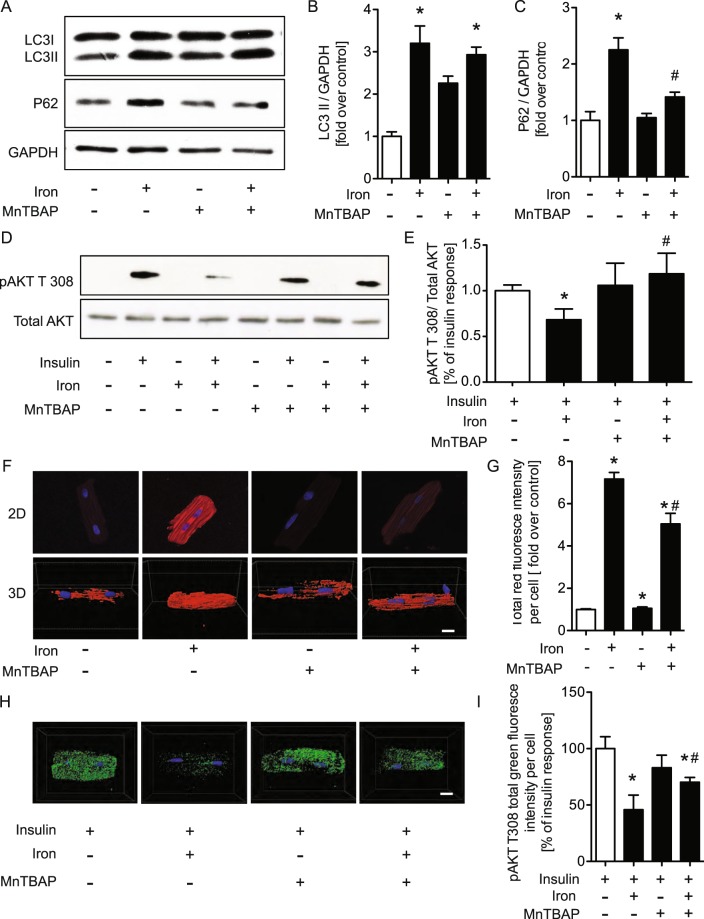
Iron induced insulin resistance via increased generation of reactive oxygen species (ROS), and enhanced by antioxidant (MnTBAP, 100 μM). H9c2 cells were treated at 4hrs with FeSO4 (100 μM) and antioxidant (MnTBAP, 100 μM). Western blots showing protein expression of LC3II, P62 and the reference protein GAPDH (**A**), followed by their quantifications (**B,C**). Insulin sensitivity was decreased in H9c2 cells as indicated by decreased phosphorylation of Akt T308; Western blots show phosphorylation of AKT T308 and reference protein, total AKT (**D**), followed by its quantification (**E**). The images were collected from two gels with same loading amounts. The blot gels shown are representatives of four different experiments (n = 4). Representative confocal images of CellROX Red assay (**F**) and phosphorylation of AKT T308 (**H**) in adult cardiomyocyte cells and their quantifications (**G,I**). *Indicates significant difference from control *p < 0.05, ^#^Indicates significant difference from iron ^#^p < 0.05, Scale bar = 20 μm.

We next evaluated whether iron-induced oxidative stress preceded reduced autophagy and Fig. 7A–C shows that iron-induced increases in the amount of p62 was significantly prevented by MnTBAP. Moreover, the ability of iron to induce insulin resistance was attenuated by using MnTBAP to reduce ROS production (Fig. 7D,E). Finally, we confirmed in primary adult rat cardiomyocytes that the amount of iron induced ROS production detected by CellROX^®^ red was attenuated by MnTBAP (Fig. 7F,G). This correlated with the ability of MnTBAP to significantly reduce the ability of iron to induce insulin resistance in primary adult rat cardiomyocytes (Fig. 7H,I).

## Discussion

Iron may play an underappreciated role in the development of insulin resistance and insulin resistance-induced heart failure. Insulin resistance and associated defective myocardial insulin signalling is an early and major factor in the development of heart failure^31^. Previous studies have suggested adverse cardiac remodeling in response to both iron or insulin overload and deficiency^32^. Numerous studies have demonstrated that increased serum ferritin is associated with insulin resistance and increased risk for diabetes^33^. We first investigated whether iron altered insulin sensitivity in IO and wt mice and we observed that the accumulation of serum and intracellular iron altered insulin resistance. To investigate mechanisms via which intercellular iron may induce insulin resistance in cardiomyocyte, we focused on oxidative stress. In the present study, we found that iron reduced insulin sensitivity via increased oxidative stress. Iron accumulation in H9c2 cells was done by treating them with iron. Using a colorimetric intracellular iron and PGSK assay, it was shown that there was significantly increased intracellular iron after treatment with iron for 1 4 and 24 hr. To assess the effect of iron overload on insulin resistance, pAKT T308 was examined in H9c2 cells after treatment to induce iron overload conditions by Western blotting. We observed the same effect of iron in neonatal and adult cardiomyocyte cells and the data showed that reduced phosphorylation of AKT T308 at 1,4 and 24 hr by Western blotting and immunofluorescence.

Previous work has included evidence that iron-induced oxidative stress is a critical driver in the pathogenesis of myocardial tissue injury and progressive development of iron overload cardiomyopathy^34^. Excess iron promotes oxidative stress via the Fenton reaction, which plays a key pathogenic role in myocardial injury and heart failure^35,36^. Although many studies have described positive relationships between modulation of adipokines and insulin resistance, much importance has been placed on iron-mediated oxidant stress and the role it plays in the development of insulin resistance^37^. Our study suggested that using CellROX Deep Red assay we also observed that iron increased generation of ROS and that using anti-oxidant (MnTBAP) to attenuate ROS production reduced the iron effect.

To investigate another novel mechanism via which iron may induce insulin resistance we focused on autophagy. This important intracellular degradation machinery captures cytoplasmic components for lysosomal degradation and recycling^38^ and previous evidence suggests that reduced autophagy occurs in the failing heart and is directly stimulated as a self-protective response in response to ischemia/reperfusion^39,40^. Autophagy is typically upregulated in times of stress, for example during ischemia/reperfusion, pressure overload and cardiac toxicity induced by chemicals such as the anthracycline doxorubicin^41^. Previous *in vivo* studies also have shown that the expression of multiple autophagy-related genes was altered in iron overload cardiomyopathy, possibly contributing to cardiac diastolic dysfunction^42^. Therefore, we used various assays designed to observe dynamic process of autophagic flux in order to carefully characterize the effect of iron. We observed that iron caused a significant upregulation of phosphorylation of ULK1 at Ser757 and LC3II. We also examined autophagic flux by testing p62 expression and found this was elevated by iron and chloroquine. LC3-II and p62 data collectively suggest iron attenuates flux. Cyto-ID is a fluorescent marker of autophagosomal puncta^43^ and both iron or chloroquine increased total amount of autophagosomes. We used the Click-iT® HPG Protein Synthesis Assay kit to gauge protein synthesis and it can also be used to monitor protein degradation^28^. Based on our data, we concluded that iron inhibited protein degradation, as did chloroquine, which is again in keeping with the phenomenon of reduced autophagy.

We have further examined the mechanistic role of autophagy in cardiac insulin sensitivity. To do so we used autophagy-deficient cell model by overexpressing a dominant-negative Atg5 mutant^30^. Our data suggested that iron overload and autophagy deficiency both induced insulin resistance and autophagy is a very important role of regulation insulin resistance in cardiomyocyte. Numerous studies suggested that dysregulation of autophagy leads to increases in oxidative stress^44,45^. For example, inhibition of autophagy by lysosome inhibitor chloroquine or the cathepsin D inhibitor pepstatin A increased ROS^46,47^. Additionally, the disorder of initiation of autophagy leads to accumulation of ubiquitinated proteins, induced ROS and elicited mitochondrial dysfunction^48^. Reduction of Atg5 and Atg10 expression exacerbated starvation-induced ROS^44^. Importantly, since our data indicated that inhibition of autophagic flux induced insulin resistance, we used AdipoRon, an adiponectin receptor agonist as an activator of autophagy^49^ and found that improved autophagy correlated with increased insulin signalling as well as attenuation of ROS production. The precise molecular mechanisms responsible for the connection between reduced autophagy and insulin resistance must be further explored and may include indirect effects via altering levels of oxidative or endoplasmic reticulum stress or a direct effect via autophagosomes playing a scaffolding role in formation of specific signaling complexes^49^. Overall, our study provides important new knowledge on a molecular mechanism contributing to insulin resistance. As a consequence, we propose that iron overload, as commonly found in type 2 diabetes, obesity and inflammatory conditions, leads to heart failure by attenuating levels of autophagy, inducing ROS and consequently insulin resistance.

## Conclusions

In conclusion, our study indicated that iron induced insulin resistance in cardiomyocytes and this involved regulation of the crosstalk between autophagy and oxidative stress. Further studies will investigate mechanisms via which iron regulates cardiac remodeling and their physiological significance. We anticipate that our findings will provide new knowledge relevant to current diagnostics and therapeutics related to altered iron status in clinical settings.

## Materials and Methods

### Cell culture

The detailed method of growing H9c2 cells was based exactly as in a previous publication^18^. For assays specific to this project, cells were grown on cover slips where required and treated with or without FeSO_4_ (100 μM) in DMEM with 0% FBS at approximately 60% confluency for 1, 4 and 24 hr. This dosage of iron is the range that is typically used by researchers in the field to modulate the activity of iron regulatory proteins (IRP1 and IRP2) and trigger homeostatic responses to iron loading^31^.

### Reagents

Apocynin (73536), FeSO_4_ (Ferrous Sulfate Heptahydrate, SIGMA-310077), MnTBAP (Mn (III) tetrakis (4-benzoic acid) porphyrin, 475870), Allopurinol (A8003) DCF-DA (D6883) were all from Sigma Aldrich. AdipoRON hydrochloride (5096) was from Tocris a biotechne brand and CellROX Deep Red Reagent (C10422) and Click-iT HPG Alexa Fluor 488 kit (C10428) were from Thermo Fisher Scientific. SkQ1 [10-(6′-plastoquinonyl) decyltriphenylphosphonium] provided by Mitotech. Cyto-ID^®^ autophagy detection kit (ENZ-51031) was from Enzo Life Sciences Inc and Magic Red Cathepsin-B Assay (938) from ImmunoChemistry Technologies LLC.

### Isolation and culture of primary adult and neonatal rat cardiomyocytes

Adult Wistar male rats (age 6 to 10 wk) were used according to protocols approved by the Animal Care Committee at York University. Adult^50^ and neonatal^51^ primary cardiomyocytes were isolated and cultured exactly as previously described by us.

### Generation of H9c2-ATG5K130R cells

To generate H9c2 cells stably over-expressing mutant ATG5 proteins (ATG5K130R), H9c2 cells were transduced with retroviral vector carrying pmCherry-ATG5K130R as we previously described^30^.

### Generation of a mouse model of acute iron overload

In house bred C57BL/6 (wild type, WT) mice (originally from Jackson Laboratory) were fed *ad libitum* on regular chow diet until 6–8 weeks of age and randomly separated into treatment groups. Animals were injected with iron dextran (Sigma) at 15 mg per kg via tail vein, three times, with two hour intervals (ie. 0, 2 and 4 h). The amounts of iron we used here are close to pharmacological doses of intravenous iron used for the treatment of iron deficiency anemia^52,53^ and less than that used in models of pharmacological iron overload that have been used in the past and provided important pathophysiological insights^10,54^. Tissues were harvested at 24 hour after initial injection. All animals were kept in temperature and humidity-control rooms (21 ± 2 °C, 35–40%) with a daily 12:12 h light-dark cycle in the animal care facility of York University in accordance to the guidelines of the Canadian Council on Animal Care. All study protocols were approved by the Animal Care Committee of York University.

### Serum iron measurement

Blood was collected via cardiac puncture. Serum was prepared by using micro Z-gel tubes with clotting activator (Sarstedt) and was snap-frozen at −80 °C. Serum iron was determined at the Biochemistry Department of the Jewish General Hospital (Montreal, Quebec) using a Roche Hitachi 917 Chemistry Analyzer as previously described^55^.

### Histological analysis of myocardial iron deposition

Heart tissue samples were fixed in neutral-buffered 10% formalin for 18 h and subjected to routine histological processing. Tissue sections were then stained with Perl’s Prussian Blue and counterstained with hematoxylin. Iron detection using this approach was determined by light microscopy.

### Analysis of iron content in heart homogenates and in H9c2 cells

Heart tissue homogenate samples were prepared as we described before^30^ H9C2 cells were lysed with 200ul of iron releasing agent (a freshly mixed solution of equal volumes of 1.4 M HCl and 4.5% (w/v) KMnO_4_ in H_2_O). The 24 well plates were sealed in aluminum foil and incubated at 60 °C for a duration of 2 hr. Afterward, 60ul of iron detecting reagent (2.5 M ammonium acetate +1 M ascorbic acid +6.5 mM ferrozine +6.5 mM Neocuproine: Sigma) was added to each well and left to incubate for 30 min at room temperature (RT). 280 ul of each reaction mixture was transferred to a 96 well plate and the absorbance of each well was measured at a wavelength of 550 nm using a spectrophotometer. Data was normalized by the control treatment without iron.

### Analysis of intracellular iron with the PG-SK fluorescent probe

H9C2 cells were treated with 100 uM of FeSO_4_ for 1, 4 and 24 hour durations 30 minutes after loading with 3uM of PG-SK diacetate (Sigma). Coverslips were washed 4 times with 0.5 ml PBS++, fixed by covering coverslips with 10% formalin solution for 30 min, washed again 3 times in PBS++, quenched by covering coverslips in 1% glycine solution for 15 minutes, washed once more 3 times in PBS++ and transferred onto slides using DAPI Mounting Media for fluorescence nuclear staining and ProLong antifade gold standard reagent (in a 1:2 ratio respectively). Prepared slides were viewed via laser scanning microscopy. PG-SK fluorescence was viewed using FITC and quantified by average fluorescent intensity per cell by dividing the total green fluorescence by cell number using ImageJ software.

### Western blotting

Heart tissue was snap frozen and pulverized with mortar and pestle in liquid nitrogen. The powdered tissue was then suspended in lysis buffer exactly as we previously described^56^. H9c2 cells were grown to 90% confluency in 6 well plates. The detailed method was based on a previous publication^18^. The following primary antibodies were used in this study: pAkt T308 (4026), Total Akt1 (9272), LC3B (2775), P62 (23214), Beclin-1 (3738), pmTORC1 S2448 (2971), pULK1 S757 (14202), pAMPK T172 (2535), p70S6K T389 (9234) and GAPDH (2118) were purchased from Cell Signalling Technology. pIR beta Y972 and pIRS-1 Y612 (44816) were purchased from Life Technology. Atg7 (376212) was purchased from Santa Cruz Biotechnology. The following secondary antibodies were used; anti-rabbit IgG HRP-linked antibody and anti-mouse IgG HRP-linked antibody from Cell Signalling Technology.

### Analysis of glucose uptake

To determine glucose uptake, H9c2 cells were seeded in 24-well plates and treated with or without FeSO_4_ (100 μM) for periods of 1, 4 and 24 hr. Where indicated insulin was used at 100 nM for 10 min. Subsequently, glucose transport was assayed essentially as we previously described^57^ and results were calculated as pmol of glucose uptake per min per mg protein.

### Detection of ROS using CellROX® deep red or DCF-DA

CellROX® deep red oxidative stress reagent (Life Technologies) which is non-fluorescent while in a reduced state and upon oxidation exhibits strong fluorogenic signal was used. H9c2 cells were seeded in 6-well plates, and treated 100 uM of FeSO4 for up to 4 hr. CellROX® deep Red was added in each well at 10ug/mL and incubated for 15 min. Cells were harvested and fixed with 4% PFA prior to analysis with confocal microscopy taken using 40 or 60X objective (LSM 700), or run in flow cytometer (Gallios ™, Beckman Coulter Inc.) to analyze red fluorescent signal intensity in each cell within a population of 100,000. Where indicated, H9C2 cells were treated 100 uM of FeSO_4_ and with various concentrations of ROS inhibitors. Iron (Ferrous Sulfate Heptahydrate, FeSO_4_), MnTBAP (Mn (III) tetrakis (4-benzoic acid) porphyrin), Apocynin, and Allopurinol were all from Sigma Aldrich. SkQ1 [10-(6′-plastoquinonyl) decyltriphenylphosphonium] provided by Mitotech. In using DCF-DA, cells were incubated for 4 hr with 20 μM DCF-DA (Sigma Aldrich) which is activated by ROS to generate a highly fluorescent 2′7′-dichlorofluorescein (DCF) molecule. DCF fluorescence was measured at 490/530 nm using a Synergy H4 multimode plate reader (BioTek).

### Cyto-ID^®^ Green and Magic Red lysosomal cathepsin B activity for autophagy detection

The Cyto-ID^®^ Green autophagy dye was prepared following the protocol from the manufacturer (Enzo Life Sciences Inc.). Magic Red assay was performed exactly as we have described before^18^. Confocal images were taken using 40 or 60X objective (LSM 700).

### Generation of 3D confocal images and time-lapse image acquisition

Images of the whole field were captured in 2D as well as in 3D (>100 z- stacks). These datasets were then loaded into IMARIS software (Bitplane Inc.) and 3D images generated. Video acquisitions were performed using a Nikon A1R confocal laser scanning microscope system (Nikon Corp., Tokyo, Japan). Video acquisition at a speed of 30 frames per second was performed for the indicated times, followed by time-lapse imaging every 2 minute up to 4 hr after Iron treatment.

### Metabolic Labeling of Proteins Synthesis assay

Click-iT® HPG Protein Synthesis Assay kit (Invitrogen) according to manufacturer’s instructions. Briefly, H9C2 cells were treated with 100 uM of FeSO_4_ for 4 hr durations 30 minutes after loading with 50 μM Click-iT^®^ HPG. Cell were washed 1 time then proceed to 15 min fixation with 10% formalin solution and 20 min permeabilization with 0.5% Triton X. Remove the permeabilization buffer and wash cells twice 3% BSA in PBS. Remove the wash solution and add cocktail to each well and mix well and incubate for 30 min at room temperature, protected from light. Remove the reaction cocktail and wash once with Click-iT^®^ reaction rinse buffer. Remove the Click-iT^®^ reaction rinse buffer and staining with DNA Staining. Scan plate using automated imaging platform with filters appropriate for DAPI/ Hoechst and FITC for Alexa Fluor^®^ 488.

### Statistical analyses

Data was presented as mean ± SEM. Statistical significance between treatment groups were calculated using the unpaired Student t test when comparing 2 groups. For comparisons of more than 2 groups, One Way ANOVA followed by Dunnett’s post-test and Two Way ANOVA with Bonferroni post-test were performed to adjust multiple comparisons. *P* value < 0.05 was considered statistically significant.

## Supplementary information

Real time video ROS production

1 - Western blot
