# Author Correction: Iron induces insulin resistance in cardiomyocytes via regulation of oxidative stress

**DOI:** 10.1038/s41598-020-58752-7

**Published:** 2020-01-30

**Authors:** Hye Kyoung Sung, Erfei Song, James Won Suk Jahng, Kostas Pantopoulos, Gary Sweeney

**Affiliations:** 1 Department of Biology, York University, Toronto, Ontario Canada; 2 Lady Davis Institute for Medical Research and McGill University, Montreal, Quebec Canada

Correction to: *Scientific Reports* 10.1038/s41598-019-41111-6, published online 15 March 2019

This Article contains an error in Figure 4A, where the incorrect image was used for the control treatment of H9c2 cells at 1 hour. The correct Figure 4 appears below as Fig. 1.

**Figure 1:**
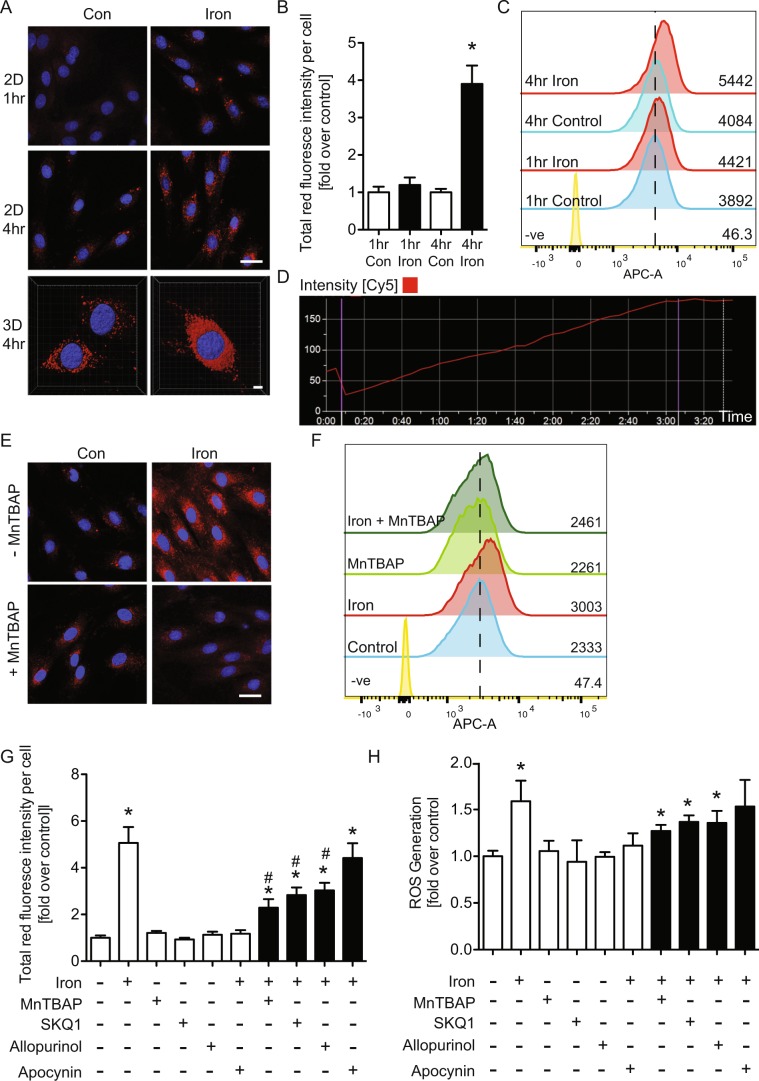
.

