# Supplementary material for: Iron induces insulin resistance in cardiomyocytes via regulation of oxidative stress
Source: Sci Rep. 2019 Mar 15;9:4668. doi: 10.1038/s41598-019-41111-6 (PMC6420583; doi:10.1038/s41598-019-41111-6)

Original article

**Iron induces insulin resistance in cardiomyocytes via regulation of oxidative stress.**

Hye Kyoung Sung^1^, Erfei Song^1,^ James Won Suk Jahng^1^, Kostas Pantopoulos^2^ & Gary Sweeney^1#^

^1^Department of Biology, York University, Toronto, Ontario, Canada & ^2^Lady Davis Institute for Medical Research and McGill University, Montreal, Quebec, Canada.

Supplementary material - 1.

The detailed information of original western blot for figure 1.


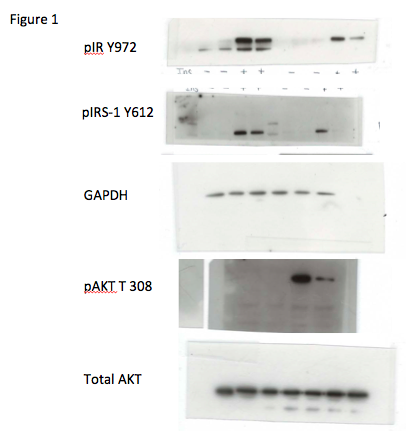


The detailed information of original western blot for figure 2.


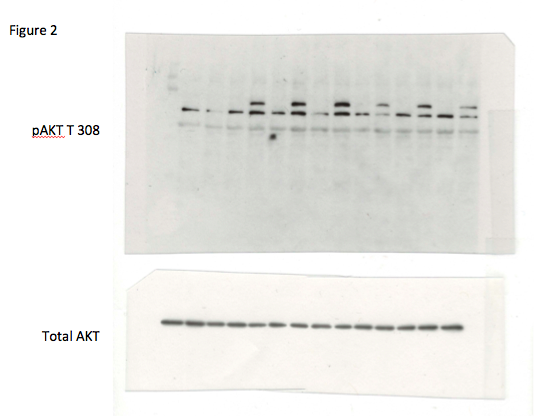


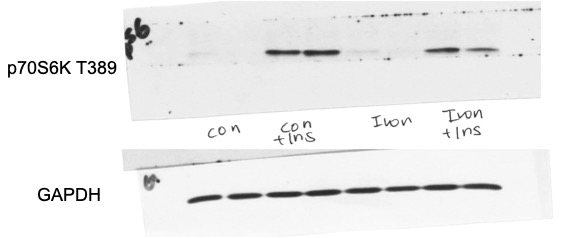


The detailed information of original western blot for figure 3.


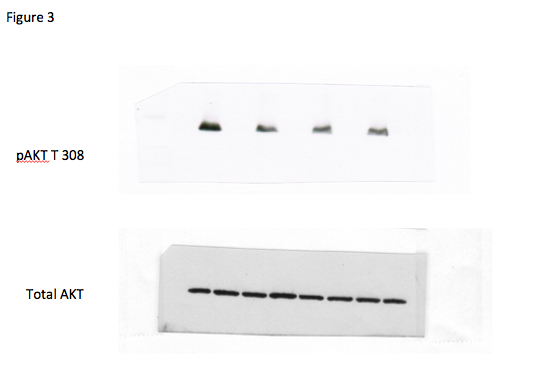


The detailed informat The detailed information of original western blot for figure 5.


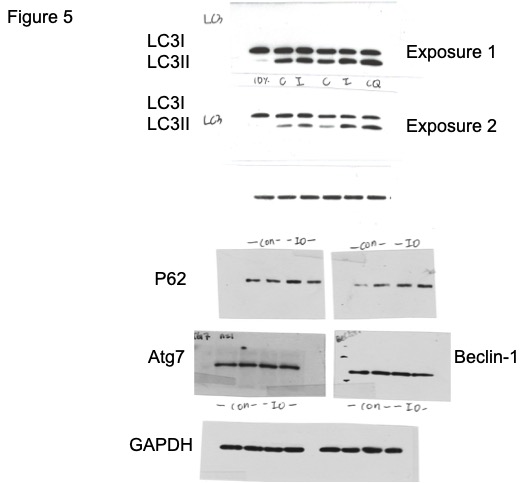

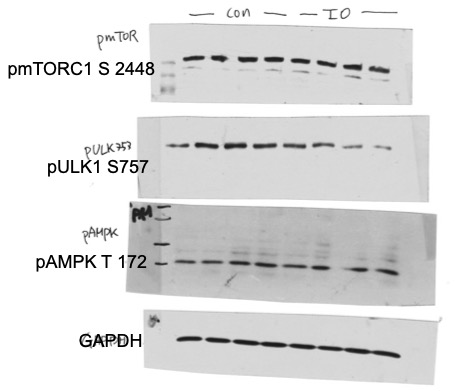


The detailed information of original western blot for figure 6.


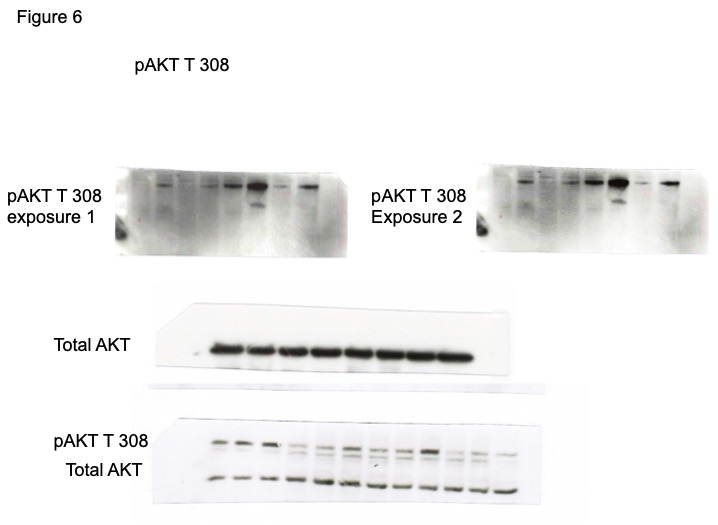


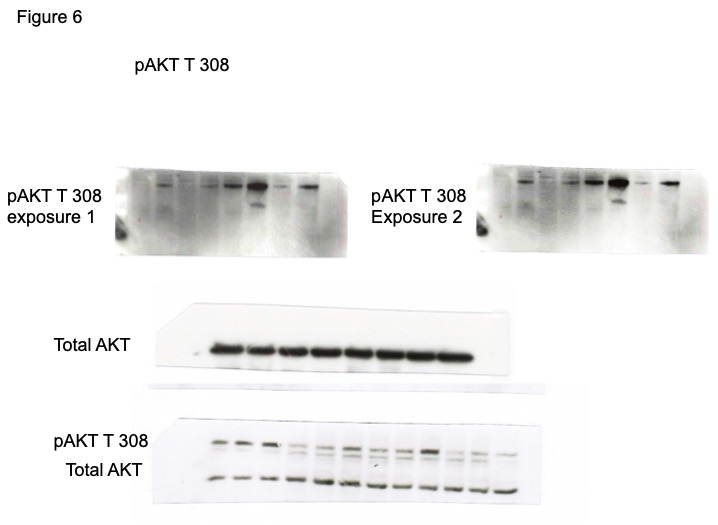


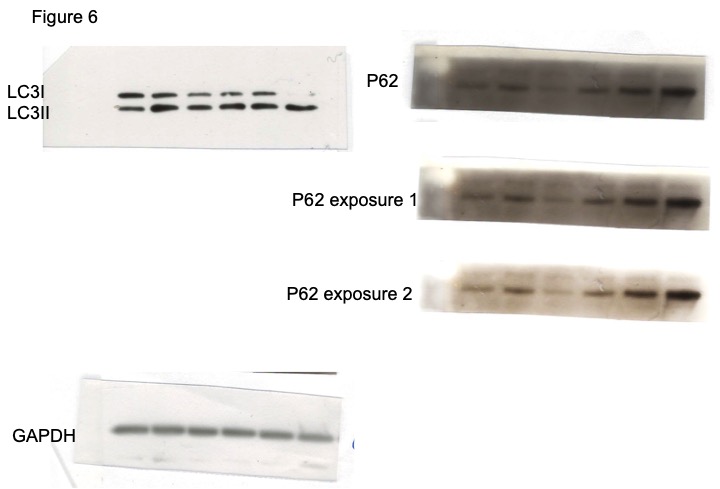


The detailed information of original western blot for figure 7.


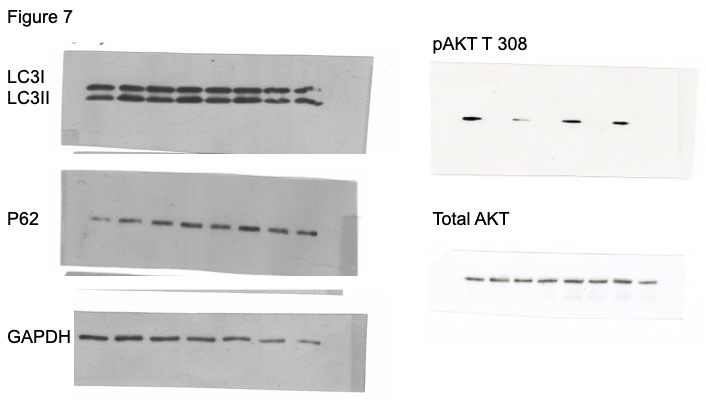

Supplement: Supplementary file 2 — 1 - Western blot [file 41598_2019_41111_MOESM2_ESM.docx]
